# Liaison Psychiatry model intervention in Switzerland

**DOI:** 10.1192/j.eurpsy.2024.88

**Published:** 2024-08-27

**Authors:** D. Georgescu

**Affiliations:** Department of Consultation-Liaison Psychiatry, Old Age Psychiatry and Neurodevelopmental Psychiatry, Aargau Psychiatric Services, Windisch, Switzerland

## Abstract

**Abstract:**

Consultation and liaison psychiatry (C-L psychiatry) in Switzerland can look back on a long tradition. It began in French-speaking Switzerland back in the 1960s and gradually spread throughout the country. Currently, C-L services are present throughout the country, although they differ greatly in terms of their services and dimensions. University hospitals and larger cantonal hospitals have extensive and differentiated services, while smaller hospitals in peripheral regions only offer basic services. There are also major differences in the financing models, which are decisive for the range of services offered. The question of funding, which has not yet been resolved satisfactorily despite various models and strategies, including at national level, is highly relevant for the further development and even the continued existence of C-L services. The introduction of the subspecialization in C-L psychiatry in 2010 and the lively training and CPD activities are of great importance for quality of the delivered services.

**Disclosure of Interest:**

None Declared

